# Orthotics and taping in the management of vertebral fractures in people with osteoporosis: a systematic review

**DOI:** 10.1136/bmjopen-2015-010657

**Published:** 2016-05-04

**Authors:** Victoria A Goodwin, Abigail J Hall, Emily Rogers, Alison Bethel

**Affiliations:** 1 NIHR CLAHRC South West Peninsula, University of Exeter, Exeter, UK; 2 Royal Devon and Exeter NHS Foundation Trust, Exeter, UK

**Keywords:** osteoporosis, vertebral fracture, orthotics, systematic review

## Abstract

**Objective:**

To establish the current evidence base for the use of orthotics and taping for people with osteoporotic vertebral fracture (OVF).

**Design:**

Systematic review of quantitative and qualitative studies.

**Data sources:**

Medline, Medline-In Process, EMBASE, AMED, CINAHL, PEDro, TRIP, EThOS, ProQuest Dissertations and Theses and Cochrane (CDSR, DARE, CMR, HTA, EED) plus Cochrane Central, UK Clinical Research Network portfolio, Controlled Clinical Trials register and the Australian and New Zealand Clinical Trials register.

**Eligibility criteria for selecting studies:**

All study designs were considered if they reported in English and evaluated the impact of using an external support, such as a spinal brace, orthosis or postural tape, with adults with OVF. All outcomes were considered.

**Results:**

Nine studies were included comprising two parallel-group randomised controlled trials, four randomised cross-over trials, two before-after (single arm) studies and a parallel group observational study. No qualitative studies were identified. A wide range of outcomes assessing impairments, activities and participation were assessed but the findings were mixed. The quality of studies was limited.

**Conclusions:**

The current evidence for using orthotic devices or taping for people with OVF is inconsistent and of limited quality and therefore careful consideration should be taken by clinicians before prescribing them in practice.

**Systematic review registration number:**

CRD42015020893.

**Strengths and limitations of this study**

- The review comprised a broad search strategy, including grey literature, to maximise the capture of all of the relevant literature.
- No qualitative studies have been undertaken to establish the experiences of using people with osteoporotic vertebral fractures using orthotics or braces.
- The included studies were generally of unclear risk of bias using the Cochrane tool.

## Introduction

Symptomatic osteoporotic vertebral fractures (OVF) affect 1 in 6 women and 1 in 12 men during their lifetime.^1^ In Europe the annual incidence of OVF is 10.7 and 5.7 per thousand in women and men, respectively.^2^ In the USA, 700 000 people are reported to have an OVF each year,^3^ however, these figures may underestimate the actual size of the problem as most OVF go unreported and, therefore, undiagnosed.^4^ With the prevalence of osteoporosis predicted to increase by 2021, a rise in associated fractures is also likely.^5^

OVF have personal, societal and economic costs.^6^ Patients experience severe pain in the acute phase but also up to 2 years post-fracture.^1^ ^7^ Increased disability and depression, and a reduced quality of life are also reported.^8^ Up to a fifth will suffer a subsequent vertebral fracture within a year^9^ and there is an increased risk of mortality.^10^ The use of primary care services has been found to be 14 times that of the general population,^11^ with 8% of people with OVF requiring hospitalisation.^12^ In the USA, the management OVF has an annual estimated cost of $13.8 billion.^1^

Guidelines for the non-medical and non-surgical management of OVF are conflicting. The American Academy of Orthopaedic Surgeons (AAOS)^13^ report inconclusive evidence in relation to rehabilitative interventions whereas the Scottish Intercollegiate Guidelines Network (SIGN)^14^ recommend electrical field therapy and supervised exercise programmes. There are a variety of external support devices available including rigid thoracolumbar spinal orthoses (TLSO) or hyperextension braces that are often used in the management of OVF, however, they are not recommended in current guidelines.^13^ Bracing is reported to assist healing when worn for up to 3 months but when worn for an extended period can result in muscle atrophy, postural muscle weakness^15^ and skin problems.^16^ This said, these devices are used by the people with OVF and clinicians continue to prescribe them, although practice varies.

In view of recently published studies, this review aimed to identify and update the current evidence base for the use of orthotics and taping for people with OVF.

## Methods

We used the preferred reporting items for systematic reviews and meta-analyses (PRISMA) as a guideline for reporting this study. A predefined protocol was registered with PROSPERO (CRD 42015020893).

### Identification and selection of studies

We included primary studies that used quantitative or qualitative methods evaluating the impact of using an external support, such as a spinal brace, orthosis or postural tape, with adults with OVF. We were interested in outcomes relating to the WHO International Classification of Functioning, Disability and Health (ICF) domains of body structure and function, activities and participation. We were also interested in the experiences and perceptions of users of the external support.

We excluded studies that involved traumatic vertebral fractures, non-vertebral fractures and those involving children, reviews and opinion papers, studies published only as an abstract and those where full text was not available in English. We also excluded controlled studies where the intervention also included surgical, pharmacological and rehabilitation interventions, except where these were provided to intervention and comparator participants. For non-controlled studies, only those where the evaluation related to the orthotic device/tape were included.

The search strategy (see online supplementary file) was applied from 1980 to April 2015 to Medline, Medline-In Process, EMBASE, AMED, CINAHL, PEDro, TRIP, EThOS, ProQuest Dissertations and Theses and Cochrane (CDSR, DARE, CMR, HTA, EED). In addition we searched clinical trials databases, including Cochrane Central, UK Clinical Research Network portfolio, Controlled Clinical Trials register and the Australian and New Zealand Clinical Trials register. We also undertook forward and backward citation checking.

10.1136/bmjopen-2015-010657.supp1 Supplementary data

The search and screening process was managed using Endnote. Two reviewers independently screened titles and abstracts. Full-text papers were then screened against the eligibility criteria independently by two reviewers. Discrepancies were discussed and agreed.

### Assessment of study characteristics

Data were extracted using a prepiloted form by one reviewer and checked by a second. The data included study characteristics (study design, selection criteria, setting and sample size), funding sources, ethical approval, population (age, gender, time since OVF), intervention and comparator characteristics (nature of intervention, duration of wear, concomitant interventions and adherence), outcomes, time points of follow-up and findings.

Study quality was assessed using the Cochrane Risk of Bias tool^17^ and was extracted by one reviewer and checked by a second. The data included random sequence generation, allocation concealment, blinding of participants and personnel, blinding of outcome assessment, incomplete outcome data, selective reporting and other sources of bias. Each item was rated as low risk, high risk or unclear risk of bias and reported for individual studies and across the studies.

### Data synthesis

A narrative approach was used to synthesise the study findings due to methodological (study design, outcomes) and clinical (participant and intervention characteristics) heterogeneity. This approach enabled exploration of relationships within the data.^18^ Risk of bias was summarised for individual studies and across studies.

## Results

**Figure 1:**
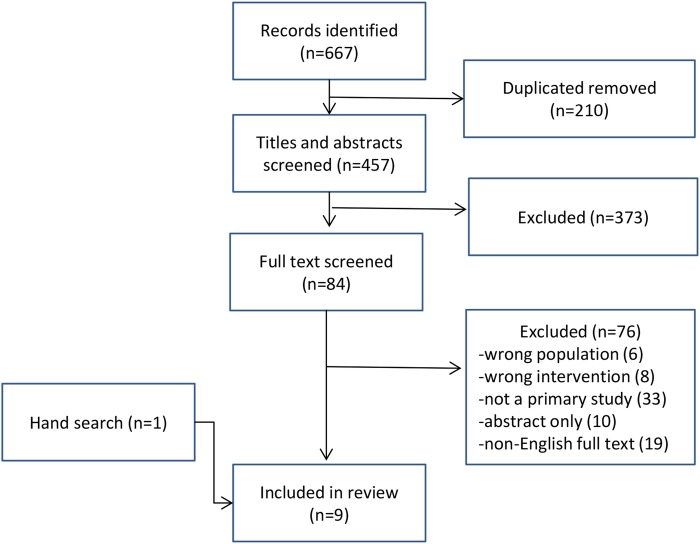
Flow chart of study selection and inclusion.

Our initial search resulted in 667 citations (figure 1) of which 84 were assessed against the selection criteria; the remainder did not meet the inclusion criteria. Nine studies were included in this review

### Characteristics of studies

The studies included 468 participants of which 404 (86%) were women. Where reported, participants had a mean (SD) age of 72.1 (7.9) years and had sustained an OVF between 3 days and 2 years previously. Six studies did not report time since fracture. The number of participants in each study varied from 13 to 108 (Mean=52). Studies took place in Europe,^19–22^ Asia^23–26^ and Australia.^27^ The majority of participants were ambulatory and community-dwelling. One study was undertaken with inpatients.^25^ Most studies took place in an outpatient setting with the setting being unclear in one study.^26^

Eight studies evaluated six different orthotic devices including rigid supports (TLSO, 3 point orthosis, plaster corset); semi-rigid supports (Spinomed and Spinomed Active); and flexible supports (soft brace) and one examined the effect of postural taping. Four studies evaluated two different types of orthotics. Controls included no brace, an alternative orthotic device (soft brace) or placebo (hypoallergenic tape). The devices were worn for between 15 min and 24 h a day (table 1). Adherence to wearing the orthotics was assessed by self-report in three studies^21^ ^22^ ^26^ although adherence was not defined. Additional data regarding individual study characteristics are reported in table 1.

**Table 1:** Characteristics of included studies

	Study design	Study participants	Sample size	Intervention	Comparator	Follow-up
Greig *et al*^27^	Crossover RCT	Community dwelling women >50; OVF<2 years; post menopause >5 years; confirmed osteoporosis	15	Therapeutic taping	Hypoallergenic tape No tape	Immediate
Kim *et al*^26^	Parallel group RCT	Age >50; acute back pain from single OVF within 3 days of minor trauma	60	Rigid TLSO for 8 weeks worn continuously except when lying down Soft brace for 8 weeks worn continuously except when lying down	No brace	12 weeks
Li *et al*^25^	Parallel group pilot RCT	Women >55; OVF T1 to T5; back pain due to OVF	51	Spinomed brace 3 h a day for 2 weeks+soft brace remainder of day	Soft brace worn all day	2 weeks
Liaw *et al*^24^	Crossover RCT	Age 65–85; confirmed OVF; severe osteoporosis	47	Rigid TLSO	No brace	Immediate
Murata *et al*^23^	Before-after study	Acute back pain <1 week; confirmed OVF	55	TLSO for at least 2 months (24 h/day except for when bathing)	–	6 months
Pfeifer *et al*^22^	Crossover RCT	Ambulatory community dwelling women >60; 1+ OVF; kyphosis ≥60^o^	62	Spinomed for 2 h a day	No brace	6 months
Pfeifer *et al*^21^	Crossover RCT	Ambulatory community dwelling women >60; 1+ OVF; kyphosis ≥60^o^	108	Spinomed for 2 h a day Spinomed active for 2 hours a day	No brace	6 months
Talic *et al*^20^	Parallel group observational study	OVF	59	Three-point orthosis Plaster corset	–	4 months
Valentin *et al*^19^	Before-after study	Women >50; receiving treatment for osteoporosis; confirmed OVF; back pain >3 months	13	Spinomed worn for 15 min/day for 2 weeks; then <2 h/day for 2 weeks; then 2–4 h/day for 8 weeks	–	3 months

OVF, osteoporotic vertebral fracture; RCT, randomised controlled trial; TLSO, thoracolumbosacral orthosis.

Two parallel-group randomised controlled trials,^25^ ^26^ four randomised cross-over trials,^21^ ^22^ ^24^ ^27^ two before-after (single arm) studies^19^ ^23^ and a parallel group observational study^20^ were included. Three studies had three study arms.^21^ ^26^ ^27^ No qualitative studies were identified. Of the four studies using cross-over design, two did not report the findings from the first treatment period.^24^ ^27^ The two studies undertaken by Pfeifer *et al*^21^ ^22^ reported that almost all participants that received the intervention during the first period refused to stop using the device for the crossover. Therefore, to maintain clarity when comparing the different intervention groups in these two studies, only the findings from the first period were considered in this review.

### Study quality

**Figure 2:**
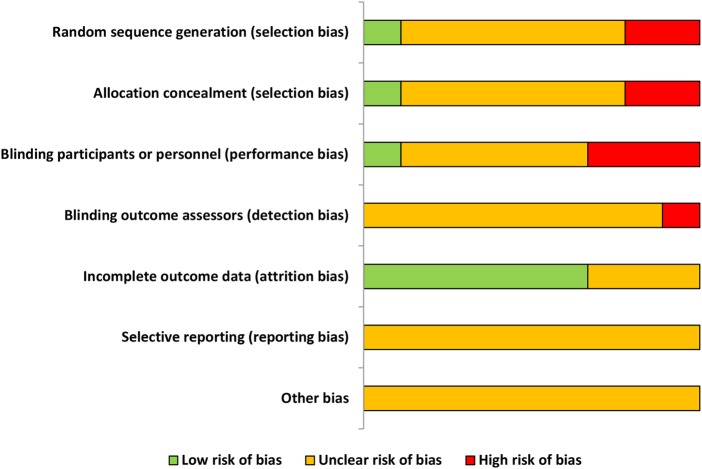
Risk of bias graph indicating proportion of studies with each judgement.

The results of the risk of bias assessment of study quality are presented in figure 2 and table 2. The reporting of studies was poor with no studies using ConSORT guidelines.^28^ Only one study reported the randomisation allocation processes. None had previously registered their studies or published a protocol. Four studies did not report ethical approvals^20^ ^22–24^ and three did not report funding sources.^20^ ^23^ ^27^ One study was termed a ‘pilot’ although the aims were stated to determine efficacy.^25^ Four studies undertook sample size calculations.^21^ ^22^ ^26^ ^27^ For the four studies using a cross-over design, additional quality assessments were made in relation to appropriate design, randomised treatment order, carry-over effect and unbiased data^29^ and these are recorded in table 2 under ‘Other’.

**Table 2:** Risk of bias summary for included studies

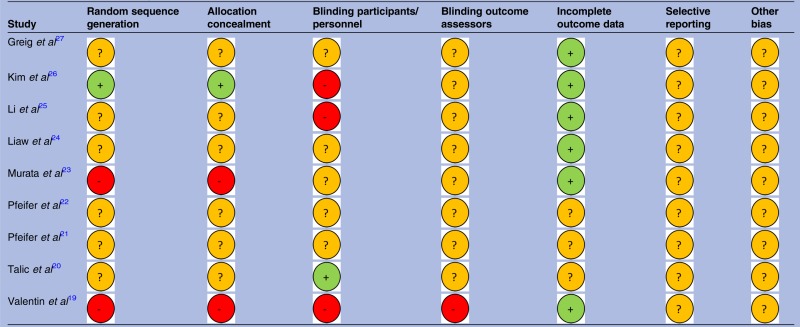

−=High risk of bias.

+=Low risk of bias.

?=Unclear risk of bias.

### Effect of the intervention

The effectiveness of orthotics and taping are reported in terms of impairments, activities and participation. Follow-up varied from immediate,^24^ ^27^ short term (<1 month),^25^ medium term (1–3 months)^19^ ^26^ and longer term (>3 months).^20–23^

#### Impairments

The majority of outcomes related to impairments such as pain,^19–22^ ^25^ ^26^ postural stability,^21^ ^22^ ^24^ back strength,^19^ ^21^ ^22^ angle of kyphosis^21–23^ ^25^ ^27^ and fracture union.^23^

Pain: Two studies^23^ ^26^ targeted those with acute fractures using TLSO or soft brace and found no benefit, whereas those studies with those with longer term fractures reported mixed findings. Pfeifer *et al* reported the Spinomed device reduced pain measured using Milner's rating scale (1=low pain to 4=very severe pain). When compared with no brace they found an Absolute Difference=−1.6 (95% CIs −2.1 to −1.1), with similar findings from an earlier study.^21^ ^22^ Valentin *et al*,^19^ however, did not find improvements in pain on a 0–10 scale (with higher scores indicting worse pain) at 3 months when using Spinomed (median difference (range)=−1 (−4.7 to 1.7); p=0.06). Li *et al*^25^ reported that Spinomed was no better than a soft brace after 3 weeks using a 0–10 pain scale (mean pain (SD) 4.0 (2.0) vs 4.5 (2.1)).

Postural stability: A range of different methods were used to assess postural stability such as a force plate,^27^ computerised dynamic posturography^24^ and a sway metre.^21^ ^22^ Each assessment method reported multiple complex components of postural stability but there were no consistent findings within or across the studies. One cross-over study^24^ examined the immediate effect of the brace or no brace on balance performance but did not appear to consider potential carry-over effects nor report the findings from the first time period so it remains unclear as to the actual effect on postural stability of the orthosis.

Back strength: Three studies assessed the use of Spinomed on isometric back strength.^19^ ^21^ ^22^ Pfeifer *et al*^21^ ^22^ reported a mean increase (SD) of 180 (152) Newtons when wearing the device and with an absolute difference of 182 (95% CI 125.1 to 238.9) Newtons compared with wearing no brace.

Angle of kyphosis: Five studies reported angle of kyphosis using a range of techniques, including an inclinometer,^27^ radiographs^23^ ^25^ and three-dimensional photomorphometry.^21^ ^22^ Postural taping was found to have immediate improvements in thoracic kyphosis when compared with placebo or no tape (Mean angle in degrees (SD) 55.3 (13.5); 57.2 (13.8); and, 58.2 (12.3) respectively; p=0.024).^27^ Li *et al*^25^ only assessed this outcome on 10/51 participants and it was unclear as to how they were selected. Two studies were unclear as to whether the findings represented an improvement or deterioration in kyphosis.^23^ ^25^

Fracture union: Murata *et al*^23^ reported at 2 months 54.7% of participants had fracture ‘settling’; with 88.7% settled at 6 months, however, the study did not have a control group and was, therefore, not possible to establish what benefit wearing the TLSO gave over ‘normal’ healing. The reporting of adverse events was poor with only one study explicitly identifying them as an outcome of interest.^20^ Talic *et al*^20^ found that plaster corsets resulted in an increased risk with four patients (16%) developing pressure sores, with no adverse events related to using a three-point orthosis.

#### Activities

The impact of orthotics on activities was evaluated using the Oswestry Disability Index (ODI),^26^ the Functional Independence measure, Elderly Mobility Scale and Modified Functional Ambulation Category,^25^ limitations in everyday life^21^ ^22^ and walking ability component of the Japanese Orthopaedic Association Back Pain Evaluation Questionnaire (JOABPEQ).^23^ Pfeifer *et al*^21^ ^22^ reported reduced disability associated with using Spinomed when compared with no brace (absolute difference −2.3 (95% CI −1.7 to −2.9),^21^ although it is unclear whether this is superior to a soft brace.^25^ Kim *et al*^26^ found no between group differences in ODI for those with an acute OVF when comparing TLSO (mean difference −1.88; 95% CI −7.02 to 9.38) or soft bracing (mean difference 2.41; −7.86 to 9.27) with no brace.

#### Participation

Participation, in relation to quality of life and well-being, was evaluated in four studies.^19^ ^21^ ^22^ ^26^ The SF36 domains were not improved by using a TLSO or soft brace when compared with no brace (Mean Physical component score (PCS) 32, 35 and 30 respectively; p=0.716)^26^ or Spinomed (median within-group difference in Physical component score=6.5, range −9.2 to 13.3; p=0.07)^19^ whereas the Hobi well-being scale did improve after wearing Spinomed (absolute difference=12.7; 95% CI 9.7 to 15.7).^21^ There was no indication that either outcome was moderated by population characteristics.

## Discussion

This review aimed to establish the effectiveness of orthotics or taping in the management of OVF. We found that the nine included studies were of limited quality and reported varied populations, interventions and outcomes. For example, no studies reported whether outcome assessors were blinded to allocation, thus the potential for detection bias is unknown. A previous review of the non-surgical management of OVF included three RCTs evaluating bracing^30^ reported medium term pain relief and reduced disability; however, studies were considered to be of low/very low quality. We found little consideration of any potential adverse events associated with bracing or taping with this population. It has been suggested that adherence to wearing orthotics is poor^31^ but despite several included studies stating adherence data was collected it was never defined and rarely reported. In addition, there is a complete absence of qualitative research involving this population and their experiences of wearing these devices which would be an essential component of any future development and evaluation. This said, in two cross-over studies,^21^ ^22^ the fact that the participants refused to stop wearing the orthotics at the point of crossover would suggest positive experiences.

We found no evidence to counter the recommendations of the American Academy of Orthopaedic Surgeons^13^ that indicated there was inconclusive evidence to support the using of bracing in the management of OVF, and that the quality of studies in this clinical area remains limited. Our findings also concur with a recent systematic review of taping that reported it was no better than placebo or no taping in terms of pain or disability for people with back pain.^32^

In terms of strengths, our study used contemporary methods for undertaking a systematic review and registered the protocol prospectively on PROSPERO. We searched an extensive range of databases, including grey literature. One limitation was the exclusion of non-English full-text papers, however, we are confident from our extensive search (that was not restricted to English) and screening process that any potential papers would have failed to meet other selection criteria and therefore would not have been included in the review. It could also be suggested that including non-randomised and non-controlled studies is a limitation. However, the purpose of our review was to establish the current evidence base, and not establish effectiveness, and therefore we considered it important to include all study designs. In addition, the use of the risk of bias tool enabled a judgement on the overall quality of the included studies, and we found that even the included randomised controlled trials were not without limitations.

## Conclusions

There is inconclusive evidence that TLSO or soft braces reduce pain or disability. Although the use of the Spinomed appears to have some benefit in terms of increasing back strength and reducing disability, it does not necessarily offer better outcomes when compared with other devices, such as soft braces. The quality of studies examining the effectiveness of orthotics or taping for the management of OVF is generally limited and therefore we would err on the side of caution when considering their use in clinical practice. Overall, there is limited evidence for the use of orthotics or taping either in the acute or long-term management of those with OVF. Further studies using high-quality methods and reporting are required to determine whether taping or orthotics are effective and cost-effectiveness.
