# Supplementary material for: Orthotics and taping in the management of vertebral fractures in people with osteoporosis: a systematic review
Source: BMJ Open. 2016 May 4;6(5):e010657. doi: 10.1136/bmjopen-2015-010657 (PMC4861088; doi:10.1136/bmjopen-2015-010657)
Supplement: Supplementary data [file bmjopen-2015-010657supp.pdf]

Database: Ovid MEDLINE(R) In-Process & Other Non-Indexed Citations and Ovid MEDLINE(R) <1946 to Present>

Search Strategy:

----

1 osteoporosis/ (36168)
2 bone density/ (42226)
3 osteoporo*.ti,ab. (54743)
4 osteopaenia.ti,ab. (104)
5 spinal fracture/ (10725)
6 (vertebra* adj3 fracture*).ti,ab. (8886)
7 fragility fracture/ (0)
8 (fragil* adj fracture*).ti,ab. (1853)
9 fractures, compression/ (1304)
10 (compress* adj fracture*).ti,ab. (3013)
11 (osteopor* adj3 fracture*).ti,ab. (10434)
12 (wedge adj fracture).ti,ab. (51)
13 kyphosis/ (4908)
14 kyphosis.ti,ab. (5401)
15 orthotic devices/ (5129)
16 athletic taping.ti,ab. (10)
17 brace/ (4500)
18 brac*.ti,ab. (74966)
19 taping.ti,ab. (1109)
20 orthotic*.ti,ab. (2224)
21 orthos*.ti,ab. (15904)
22 (postur* adj3 tap*).ti,ab. (37)
23 corset*.ti,ab. (505)
24 1 or 2 or 3 or 4 (88968)
25 5 or 6 or 7 or 8 or 9 or 10 or 11 or 12 or 13 or 14 (31311)
26 15 or 16 or 17 or 18 or 19 or 20 or 21 or 22 or 23 (97241)
27 24 and 25 and 26 (189)

****
